# Mutual Interactions between Aquaporins and Membrane Components

**DOI:** 10.3389/fpls.2016.01322

**Published:** 2016-08-30

**Authors:** Maria del Carmen Martínez-Ballesta, Micaela Carvajal

**Affiliations:** Plant Nutrition Department, Aquaporins Group, Centro de Edafología y Biología Aplicada del Segura-Consejo Superior de Investigaciones Científicas (CEBAS-CSIC)Murcia, Spain

**Keywords:** aquaporins, lipids, fluidity, micorviscosity, phospholipids, sterols, heterotetramers

## Abstract

In recent years, a number of studies have been focused on the structural evaluation of protein complexes in order to get mechanistic insights into how proteins communicate at the molecular level within the cell. Specific sites of protein-aquaporin interaction have been evaluated and new forms of regulation of aquaporins described, based on these associations. Heterotetramerizations of aquaporin isoforms are considered as novel regulatory mechanisms for plasma membrane (PIPs) and tonoplast (TIPs) proteins, influencing their intrinsic permeability and trafficking dynamics in the adaptive response to changing environmental conditions. However, protein–protein interaction is an extensive theme that is difficult to tackle and new methodologies are being used to study the physical interactions involved. Bimolecular fluorescence complementation and the identification of cross-linked peptides based on tandem mass spectra, that are complementary to other methodologies such as heterologous expression, co-precipitation assays or confocal fluorescence microscopy, are discussed in this review. The chemical composition and the physical characteristics of the lipid bilayer also influence many aspects of membrane aquaporins, including their functionality. The molecular driving forces stabilizing the positions of the lipids around aquaporins could define their activity, thereby altering the conformational properties. Therefore, an integrative approach to the relevance of the membrane-aquaporin interaction to different processes related to plant cell physiology is provided. Finally, it is described how the interactions between aquaporins and copolymer matrixes or biological compounds offer an opportunity for the functional incorporation of aquaporins into new biotechnological advances.

## Introduction

The current knowledge of membrane components is rather complete, but the physical and structural aspects of the lipid-protein interactions are still under investigation and underline the complexity of the biological membrane as a whole. Two different approaches have been established to determine the protein and lipid molecules interaction in a membrane: lipid- and protein-based approaches (Lee, 2011). Lipid-based approaches consider the intrinsic physical properties of the membrane, like fluidity, permeability, or viscosity, together with the lipid chemical composition, in regard to the interference with membrane protein function. Protein-based approaches concern the molecular aspects of the proteins themselves and their interactions with other proteins. In fact, we have to consider an intrinsic membrane protein as a different type of protein, since the comprehensive function of intrinsic membrane proteins can only be understood in conjunction with their interactions with the lipid bilayer and other membrane proteins.

Plants aquaporins (AQPs) are molecular proteinaceous membrane channels that finely control the passage of water through membranes. During the last 20 years, after their discovery, AQPs have been deeply investigated and this has highlighted the difficulty in characterizing an individual role for them. The great number of AQPs in plants, the different substrates that they transport (Li et al., 2014) and the diverse forms of molecular regulation (Yaneff et al., 2015) make the study of the physiological role of AQPs a great challenge. To this complicated landscape, we have to add the recent discovery of AQP-protein and AQP-AQP interaction, which affect the regulation of aquaporin functionality (Fetter et al., 2004; Xin et al., 2014).

Biological membranes are constituted by lipids and proteins that establish physical and chemical communication between cells and their intracellular compartments. The lipid bilayer forms a fluid matrix to hold proteins. However, this lipid bilayer has been widely reported to be more than a passive fluid since it influences many aspects of membrane proteins, including their insertion into the membrane (Marsh, 2008; Dowhan and Bogdanov, 2009), assembly into complexes (Dalbey et al., 2011; Raja, 2011; Vitrac et al., 2011), and activity (Phillips et al., 2009). On the contrary, membrane proteins can alter the physical properties of lipid bilayers – mediating, for instance, pore formation (Brogden, 2005), fusogenicity (Jahn and Scheller, 2006), and membrane bending (Graham and Kozlov, 2010; Qualmann et al., 2011). Detailed knowledge of how lipids and membrane proteins interact with each other is therefore crucial to understand the molecular machinery of biological membranes.

In this review, the regulation of AQP isoforms by the physical and chemical characteristics of the lipid bilayer is considered, together with the interaction of the isoforms with other AQP isoforms and proteins. These characteristics are also described as novel regulators of membrane intrinsic proteins (MIPs), influencing the permeability and trafficking dynamics of the membrane in the adaptive response to changing environmental conditions. The molecular driving forces resulting from the positions of the lipids around AQPs, which modulate their activity, will be discussed in terms of future challenges.

## Methodologies to Study Lipid-Protein and Protein–Protein Interaction

The structures of AQPs and specific lipid-protein interactions have, classically, been determined by electron and X-ray crystallography (Andrews et al., 2008). However, due to the high hydrophobicity of the *trans*-membrane regions of AQPs, lipid-AQP stabilization is an important challenge to these methodologies. In fact, there is only limited information about pore conformation and this was determined for only two AQPs, AQP0 (Gonen et al., 2004), and the plant AQP SoPIP2;1 (Tönroth-Horsefield et al., 2005); the mechanisms involved in their regulation were not clarified.

The use of bicelles and nanodiscs presents some advantages with respect to the use of liposomes and micelles, since they confer adequate size and stability to the lipid-protein complex (Bayburt and Sligar, 2010). In particular, protein–protein interaction, protein dynamics, and protein-binding sites are addressed by this approach, but it appears to be less effective in transport assays as a consequence of the lack of protein compartmentalization.

Although protein reconstitution in proteoliposomes has been used widely to study AQPs functionality, there is also evidence that AQPs activity may vary depending on the bilayer composition (Tong et al., 2012); this would have physiological consequences for the permeability of cell membranes. In fact, changes in the membrane lipid composition have been related to changes in AQPs permeability (López-Pérez et al., 2009).

Also, the importance of protein–protein interaction with regard to understanding biological processes is clear (Wu et al., 2009) and the functioning and regulation of AQPs in relation to these interactions have been well characterized in different cell based assays. In general, interaction between heterooligomers of AQPs is studied by co-injection of the cRNA of different PIP isoforms in a heterologous system such as *Xenopus* oocytes (Zelazny et al., 2009). However, great variation in the subsequent water permeability has been found, depending on the PIP isoforms, cRNA ratio or the experimental conditions. Complementary approaches, such as localization by confocal fluorescence microscopy, immunohistochemistry, or inhibition by cytosolic acidification, may yield more extensive information. One additional complexity regarding AQPs interaction studies is that the common methodologies have been developed for soluble proteins rather than plasma membrane (PM) proteins.

Among the methods used for protein complementation, yeast two-hybrid interaction is based on the activated expression of a reporter gene that is associated with a characteristic phenotype (Sjöhamn and Hedfalk, 2014). The interactions of AQPs with bacterial and oomycete effectors were described using this methodology (Mukhtar et al., 2011). Constructs with the transcription factor and the interacting proteins allow *in vivo* protein assembly. The method is able to detect tenuous linkages but a high rate of false positives is usual. Co-precipitation assays combined with immunodetection are feasible alternative tools for the determination of protein–protein interaction (Ciruela, 2008). Co-immunoprecipitation may discern the reciprocal actions of the different protein subunits that form a protein conglomerate. However, the main difficulties of this methodology are the cost and the time that is consumed during the design and preparation of the highly specific antibodies needed to bind to the complexes that include the bait protein (Miernyk and Thelen, 2008).

Photobleaching fluorescence resonance energy transfer (FRET) has been applied to demonstrate the physical interaction between the maize AQP isoforms Zm-PIP1s and Zm-PIP2s (Zelazny et al., 2009) as well as between Zm-PIP2;5 and the SNARE protein SYP121 (Besserer et al., 2012). Another technically demanding method is the use of bimolecular fluorescence complementation (BiFC). BiFC has several advantages in the study of protein–protein interaction (Horstman et al., 2014) and, in combination with fluorescence detection, represents a useful tool for the purification of the intact complex (Murozuka et al., 2013), especially when the proteins have a low affinity for each other. The use of BiFC may maintain the integrity of the complex formed *in vivo* and ensure that the protein targets are localized in their native cellular compartment. In addition, the fluorescence (with GFP or YFP proteins) can be traced during the solubilization or purification steps, which improves the methodology. By contrast, the cellular expression of the fluorescence particles (GFP or YFP) does not always lead to an effective fluorophore; also, protein–protein interaction may impede the correct reconstitution of the fluorescent protein (Citovsky et al., 2008).

Thus, the study of protein–protein interaction partners is a young discipline, while there are a great number of reports concerning the functional and structural information of purified AQPs. The identification and analysis of these proteins which form complexes with other membrane proteins are still a major challenge, but the large number of interacting partners that affect AQP regulation makes this discipline a promising tool in cell biology that can provide a way to answer novel scientific questions.

## Protein–Protein Interactions

### Aquaporin–Aquaporin Interaction

Protein interactions, ranging from the formation of stable complexes within the cell to transient complexes involved in cell signaling pathways, determine protein function. Membrane proteins are basic elements of the cell, allowing the transport of molecules across the membrane and communication with the external environment. Among them, the AQPs are transmembrane channels – organized in highly conserved tetrameric structures in the cell membranes – which facilitate the passage of water and/or small solutes (Maurel et al., 2008). The function of AQPs is controlled by physiological signals as well as the interactions between different AQP monomers or binding with other proteins.

The role of hetero-molecular AQP interactions has been described in plants as a mode to control physiological mechanisms (Maurel, 2007). An interesting point of view is that AQP isoforms can act on other AQP isoforms, which could be of remarkable importance in tissues where multiple isoforms are expressed. Heteroligomerization between PIP1 and PIP2 has been shown to modify the characteristics of water channels in plant cells or *Xenopus laevis* oocytes. Thus, co-expression of PIP1 with PIP2 was necessary not only to increase PIP1 water permeability (Fetter et al., 2004; Otto et al., 2010), but also for its displacement from the endoplasmic reticulum to the PM (Zelazny et al., 2007, 2009).

**FIGURE 1:**
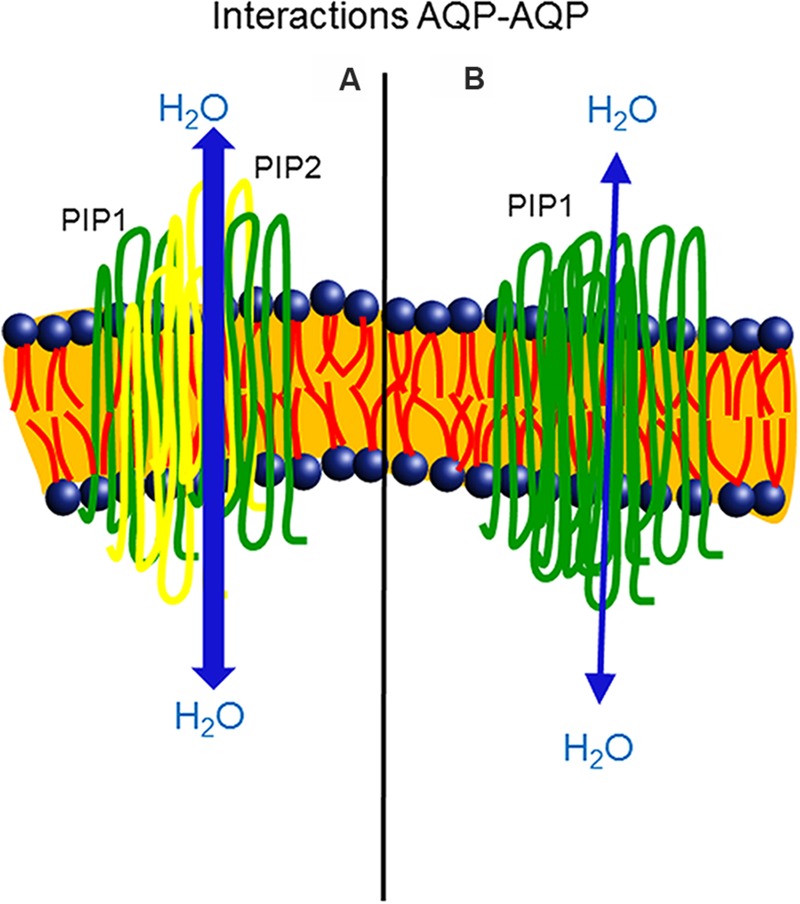
**Heteroligomerization of two PIP1 monomers (green color) and two PIP2 monomers (yellow color), isoforms of aquaporins.** The co-expression of PIP1 and PIP2 enhanced water permeability across the plasma membrane of *Xenopus* oocytes **(A)**. Homotetramer assembly of PIP1 monomers (green color) resulted in a lower capacity to transport water in the membrane **(B)**, in comparison with the heterotetramer.

The study of the structural basis for heteroligomers formation is still a challenge. In maize, a conserved cysteine (Cys) residue in the loop A of PIP1 and PIP2 proteins has been identified as responsible for the formation of a covalent disulfide bond between the monomers of these two subfamilies. Although this Cys did not directly regulate the activity or trafficking of the two PIPs, the residue increased the oligomer stability under unfavorable conditions, hence controlling PIP abundance in plant cells (Bienert et al., 2012). However, in *Beta vulgaris*, BvPIP2;1 was not able to translocate BvPIP1;1 to the PM after the co-expression of both isoforms, whereas BvPIP2;2 was assembled with BvPIP1;1. Differences in the heteroligomerization of the PIPs were attributed to differences in the functionality of loop A (Jozefkowicz et al., 2013). Also, recently, Yaneff et al. (2015) postulated that the abundance of PIPs transcripts regulates the PIP1-PIP2 associations, providing specific combinations of PIP1-PIP2 isoforms when plants are exposed to different environmental conditions and conferring on the PIP1-PIP2 pair a role as a functional unit in the related physiological processes (**Figure 1**).

Less studied are the interactions between TIP isoforms and there is a lack of information about the function of their association. The putative interactions between TIPs isoforms from *Arabidopsis* have been studied using heterologous expression in yeast (Murozuka et al., 2013). The authors described not only a strong interaction between the TIP1;2, TIP2;1, and TIP3;1 isoforms but also the assembly of TIPs with PIP2;1. Although PIPs were localized in different membranes when compared with TIPs (Maurel et al., 2008), some TIPs were present in the PM (Wienkoop and Saalbach, 2003; Gattolin et al., 2011). In the same way, it has been reported that the form of heterotetramers assembly may condition physiological processes related to plant development. In onion cells, the HvTIP1;2 and HvTIP3;1 isoforms interacted, forming a heterotetramer which increased water permeability in the cells of late ripening seeds. A high level of water permeability was induced in comparison with the expression of HvTIP1;2 homotetramers alone. Thus, tight control of the water status of the cells by HvTIP3;1 was achieved in the seed development and desiccation stages (Utsugi et al., 2015).

In cotton, fiber elongation was found to depend on PIP2s and specific PIP2 isoform combinations markedly enhanced the water permeability in *Xenopus* oocytes (Li et al., 2013). Also, in the halophyte, *Thellungiella halophila*, ThPIP1 interacted with ThPIP2 and a non-specific lipid-transfer protein 2, suggesting a role of ThPIP1 in the response to the external signals that trigger multiple physiological processes when plants are exposed to salinity (Qiang et al., 2015).

Therefore, although the formation and dissociation of the heterotetramers of MIPs represent a mechanism for dynamic changes of the membrane permeability, further studies are necessary to discern these functions of the heterotetramers in plants.

### Aquaporin Interaction with other Proteins

Hetero-complexes with AQPs, regulating different biological processes, have been described in several organisms. In humans, 70% of the AQPs interactions are comprised by the C-terminus (Sjöhamn and Hedfalk, 2014), which is the most divergent sequence in the AQP family and frequently appears as the main site of regulation. However, in plants, these interactions are emerging as new mechanisms that determine AQPs functions.

The interaction of AQPs with other proteins or nucleic acids involved in osmotic adjustment processes has been described. Xin et al. (2014) showed that transgenic *Arabidopsis* plants expressing SlTIP2;2 possessed enhanced tolerance of salinity. In addition, yeast two-hybrid and BiFC analysis in protoplasts determined the physical binding between SlTIP2;2 from tomato (*Solanum lycopersicum*) and the UDP-galactose transporter (Xin et al., 2014).

The functionality of the AQP TsMIP6, from the halophyte *T. salsuginea*, was characterized in transgenic rice (*Oryza sativa*). The expression of this gene was enhanced under salinity (Sun et al., 2015). In addition, TsMIP6 specifically interacted with a member of the glycoside hydrolase family in yeast cells. The authors suggested that the relationship between TsMIP6 and the hydrolase could mediate the osmotic balance in the plant cells and that TsMIP6 could also be involved in the transport of solutes that participate in the maintenance of the osmotic potential.

The pore-gating phosphorylation sites in AQPs were affected in inserted T-DNA mutants for the SIRK1 protein, an active kinase with increased activity in the presence of external sucrose (Wu et al., 2013). Thus, a direct interaction and regulation of the AQPs *via* phosphorylation by SIRK1 were implicated in this sucrose-specific osmotic response. In maize plants, a physical interaction between the PM syntaxin SYP121, a SNARE protein, and the AQP isoform Zm-PIP2;5 was described, with evidence that the SNAREs coordinated membrane trafficking and the activity of AQPs and plant K^+^ channels (Besserer et al., 2012). This mechanism of coordination may suppose an adjusted regulation of the movement of water and ions in growing cells, guard cells, or cells under drought stress.

Also, in *Arabidopsis*, autophagic degradation of PIP2;7 in the vacuole was regulated by a protein–protein interaction under drought stress. The *Arabidopsis* tryptophan-rich sensory protein/translocator (TSPO), a multi-stress regulator, interacted with the AQP isoform at the level of the endoplasmic reticulum and Golgi membranes, resulting in a decreased abundance of PIP2;7 protein in the PM that prevented cell water loss (Hachez et al., 2014b). The correct delivery of this PIP to the PM involved specific interactions with two syntaxin proteins, SYP61 and SYP121 (Hachez et al., 2014a). An additional anchoring role of PIP2;7 has been suggested by Kriechbaumer et al. (2015); the presence of the AQP in the plasmodesmata proteome and the interaction with the plant reticulum family proteins tubulate (RTNLB) may confirm the connection between the desmotubule and PM. In agreement with this idea, the heterologous expression of the OsPIP1;3 AQP of rice in yeast showed a direct interaction with the hairpin protein Hap1 from *Xanthomonas oryzae* pv. *oryzae* (Xoo), the pathogen that causes bacterial blight of rice. This may favor the virulence of plant pathogenic bacteria, with the AQPs acting as anchor elements (Ji and Dong, 2015). These interaction studies will form the basis for future research related to the AQP–protein interaction network, with implications in cellular functions and pathways.

## Membrane Physical Properties

### Membrane Fluidity

In living organisms, membranes are in the liquid-crystalline phase, in which there is considerable freedom of motion for the phospholipids. The membranes are characterized by high rotation of the C-C bonds of the fatty acyl chains and lateral diffusion of the phospholipids within the phase of the membrane. The amplitude of motion, described by an order-parameter, which describes the time-averaged disposition in space of each group of atoms in the acyl chains (Los and Murata, 2004), is inversely proportional to the fluidity (Laroche et al., 2001).

Membrane fluidity has been associated with many other processes in membranes, like protein activity. The effect of fluidity change on the function of membrane proteins such as ATPase was reported by Cooke and Burden (1990). They used xenobiotics to help elucidate the influence of fluidity on protein activity but encountered difficulties in their search for a direct relationship. Yeagle (1985) observed that high pressure decreased fluidity and stimulated ATPase activity, whereas addition of cholesterol also decreased fluidity but inhibited ATPase activity. Kitagawa et al. (1995) claimed to have demonstrated a correlation between fluidity and membrane-bound enzyme activity. Subsequently, fluidity changes have been reported to affect the activity of membrane proteins, like P4 ATPases, influencing the ability of the plant to adapt to cold stress (Gomès et al., 2000). In the same way, some investigations have shown that water transport through AQPs can be affected by membrane physical properties such as fluidity (Carvajal et al., 1996; Frick et al., 2013). These reports showed that high fluidity is related to high AQP functionality. Frick et al. (2013) concluded that increased membrane fluidity could affect the conformational state of SoPIP2;1, pushing the equilibrium toward an open conformation. Furthermore, Ho et al. (1994) reported that water in the membrane bilayer was also responsible for modifying fluidity, since increases in membrane water content increased fluidity. Thus, diffusion of water through the bilayer affects its fluidity and the rate of that diffusion depends on the fluidity, as determined by changes in lipid composition (sterols and/or fatty acid saturation). Also, recent findings indicate that membrane hydration increases the space in the acyl chains (Disalvo, 2015). Therefore, due to an increased presence of water molecules at the lipid/protein interface, a consequence of increasing unsaturation, an increase in AQP permeability has been observed (Lee, 2004). In this context, it should always be remembered that the PM is a heterogeneous entity comprising different domains, each with its own fluidity and viscosity (Marrink et al., 2009). Therefore, although it is now possible to obtain a reasonable quantification of the overall motion parameters in terms of membrane structure and mobility, and their influence on AQP functionality, they should be interpreted very carefully.

### Viscosity and Microviscosity

Viscosity, as a property of a fluid, is the internal friction a consequence of the cohesive forces between molecules (Lee et al., 1989). The term microviscosity was defined by Shinitzky et al. (1971) as the harmonic mean of the effective viscosities opposing the in- and out-of-plane rotations of the fluorescent probe. An effective definition of these terms that would allow translation of one to the other is, in fact, elusive, especially in those situations where microviscosity values are determined using steady-state fluorescence anisotropy values (Engel and Prendergast, 1981). Thus, in lipid bilayers, it is possible to define a relationship between fluidity and viscosity, both being dynamic parameters. Although studies of microviscosity in relation to AQPs functionality are very scarce, microviscosity has been reported to be involved in the alteration of the conformation of transmembrane channels, changing the activity of the proteins (Foulkes, 1998).

This property, that is dependent on the physical state of the membrane lipid bilayer and on the water in the membrane, has been related to AQPs functionality – its maximum influence being in the response of plants to stress conditions (Carvajal et al., 1998).

The fact that the microviscosity of lipid membranes is higher than that of water bestows on AQPs the critical parameter related to the nearly frictionless transport of water molecules through them. This phase has been identified recently as the possible phase transition temperature, after selecting specific activation energies and activation volumes for confined (cylindrical) geometry (Kwang-Hua, 2015). With this approach and model-based calculations, the study produced results that can be applied to the diverse fields of research related to transmembrane water transport *via* AQPs, thereby shedding further light on the microviscosity/fluidity-AQPs relationship

In a study of the microviscosity of the hydrocarbon zone in the isolated tonoplast and PM, using a fluorescent diphenylhexatriene probe, it was demonstrated that both membranes do not differ in this parameter in the phase state of their lipid bilayer (Trofimova et al., 2001). The study pointed out that the observed difference in water permeability does not depend on the state of the lipid phase and probably reflects the dissimilar functional activity of PM and tonoplast AQPs. However, the study did not take into account that the tonoplast and PM possess different amount of AQPs – that result in differing osmotic water permeabilities (Maurel et al., 2008), independently of the physical properties of the membrane.

## Lipids

### Sterols

It has always been assumed that the lipid composition of the membrane is the major factor determining its physical characteristics. The function of sterols in plants was thought to be related primarily to their ability to affect membrane structure and water permeability (Graziani and Livne, 1972). However, they can also affect the packing of the membrane bilayer, interacting with fatty acid side-chains of phospholipids and integral membrane proteins, increasing fluidity (Cooper, 2000) and bilayer water permeability (Da Silveira et al., 2003). In relation to this, beside the fact that a high sterol content increases fluidity and thereby AQPs functionality, other studies indicate that the highest local concentrations of PIP-AQPs correspond to tightly packed, sterol-enriched domains (Belugin et al., 2011). These domains are correlated with higher water permeability that probably corresponds to both AQPs and the lipid bilayer.

Also, it has been reported that an increase in the proportion of AQPs in the plant DRM (detergent-resistant PM fraction), which is rich in sterols and is considerably different from the total PM fraction, increased the osmotic water permeability of the PM at low and freezing temperatures and, hence, increased cell survival (Minami et al., 2009). Also, discoveries regarding the regulation of AQP intracellular trafficking and sub-cellular localization in response to environmental stresses, like water shortage and salt stress, revealed that sterol-rich domains are crucial in the cell surface dynamics and endocytosis of PM AQPs (for reviews see Hachez et al., 2013 and Luu and Maurel, 2013). Therefore, the interactions between AQPs and sterol-enriched domains seem to be closely related to AQP membrane functionality (Belugin et al., 2011). All these studies have contributed to the deciphering of AQP sub-cellular trafficking, which has been reported to be related to plant growth and development in *Arabidopsis* as a response to environmental changes (Boursiac et al., 2005). In fact, the content of sterols seems to be related to the higher or lower resistance to salinity that involves AQPs functionality (López-Pérez et al., 2009; Chalbi et al., 2015). High salinity usually produces an increase in total PM sterols (Silva et al., 2007; López-Pérez et al., 2009). However, the results obtained for halophytes revealed that in *Cakile maritima* the total PM sterols decreased, reducing membrane rigidity (Chalbi et al., 2015). This was related to low membrane stability, but not to water permeability. However, rather than total sterols, the stigmasterol/sitosterol ratio should be used as the parameter that indicates a swift increase in water permeability. Sitosterol has been shown to be very efficient regarding the regulation of water permeability in plants grown under salt stress (Silva et al., 2007; Basyuni et al., 2012), and it is related closely to AQP functionality (López-Pérez et al., 2009).

In the same way, the results in animals provide evidence that the sterol content in the membrane could shift the balance toward the transport of other molecules, rather than water, by AQPs. Therefore, it has been concluded that sterols, which impart mechanical stability to the membrane, reduce gas permeability through the lipid bilayer but can markedly raise the gas permeability through AQPs. In this sense, Itel et al. (2012) showed that cholesterol can decrease membrane CO_2_ permeability by two orders of magnitude in phospholipid vesicles and intact cells. However, the permeability of CO_2_, and possibly other gasses, through AQP1 appeared to be increased by three orders of magnitude. Also, studies with kidney cells revealed that cholesterol depletion reduced the diffusion coefficient of AQP3 but not that of AQP5 (Koffman et al., 2015), suggesting that only a subset of AQPs may be associated with lipid rafts and regulated by cholesterol.

### Phospholipids

The phospholipid composition, in terms of the head-group and acyl chains, also seems to be related to membrane physical properties which influence the activity of proteins. In studies of the PM composition of wheat plants grown under drought stress, it was observed that the phosphatidylcholine/phospha- tidylethanolamine ratio and the level of unsaturation of the fatty acyl chains increased. It was suggested that this produced a more fluid lipid matrix in order to preserve the physiological function of the lipid bilayer (Vigh et al., 1986). Similar findings, plus an increase in free sterol abundance, were observed in water-stressed sunflower PM (Navarri-Izzo et al., 1993). However, after the discovery of AQPs, it was found that the adaptation to stress was dependent on the AQPs rather than on lipid composition, although the phospholipid composition could modulate AQP functionality through effects on the physical properties of membranes, such as fluidity (Carvajal et al., 1996).

Molecular dynamic simulations and crystallographic refinement to determine the localization of DMPC (dimyristoylphosphatidylcholine) lipids around lens-specific animal water channel aquaporin-0 (AQP0) showed that the positions of the constrained lipids in the 2D crystals are defined by the acyl chains rather than the head groups (Aponte-Santamaría et al., 2012). Furthermore, the positions of these lipids are influenced greatly by the local mobility of the protein, whereas specific hydrogen bonds play a secondary role. Therefore, AQPs follow the general mechanism in which membrane proteins diffuse laterally, associated with the two layers of lipids, with the positions of the lipids in the first solvation shell being modulated also by irregularities in the protein interface (Lindahl and Sansom, 2008).

## Interaction of Aquaporins with Copolymers

Supported biomimetic membranes (SBMs) have attracted much interest as enabling components in promising applications such as water purification (Kaufman et al., 2010; Zhong et al., 2012). Hence, AQPs incorporated into SBMs created a potential high-performance water purification membrane with high water permeability and reliable ion rejection, exceeding the yield of commercial polymeric membranes (Kumar et al., 2007). However, two critical aspects should be borne in mind for the design of SBMs containing AQPs (Xie et al., 2013): AQPs must be highly selective to water and show great transport capability, and there is a need for high strength of the membrane matrix. For the first requirement, the influence of the membrane composition is very important. The big challenge is to characterize all AQPs and determine the most suitable lipid surroundings, to maximize water transport. Also, the preparation of stable, functioning SBMs and their integration and interfacing with an appropriate, robust supporting structure still remain highly challenging. Recently, SBMs were successfully prepared on silica using a positively charged, single-chain bolalipid GLH-20 (Kaufman et al., 2013). Unlike common SBM precursors (vesicle-forming lipids), GLH-20 in solution forms micelles, rather than vesicles, which spontaneously form a stable and contiguous supported SBM with a low defect rate.

In the same way, Xie et al. (2013) designed and fabricated a stable and functional polymeric membrane embedded with nano-sized AQP vesicles, through an innovative yet simple and easy-to-implement method based on an AQPz-incorporating biomimetic membrane and surface imprinting polymerization technology. They concluded that the AQPz functionally incorporated into polymer vesicles exhibits high mechanical strength and stability during the water filtration process. Also, the micro-batchwise methodology has been reported to be suitable for the functional reconstitution of rice AQP and other membrane transport proteins (Scalera et al., 2014).

Therefore, the possibility of mimicking biological membranes and the challenges of fabricating separation devices based on such biomimetic membranes have both increased in recent years, which offer an opportunity for the functional incorporation of AQPs in new technologies.

## Concluding Remarks

**FIGURE 2:**
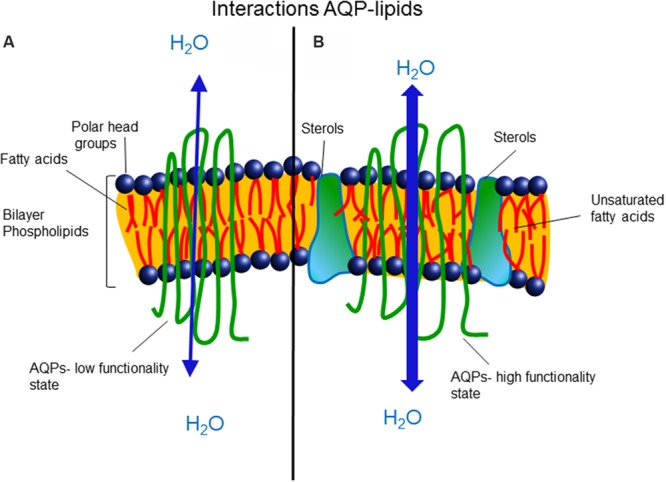
**(A)** Saturated hydrocarbon fatty acids are related with low membrane fluidity, high microviscosity, and low aquaporin functionality. **(B)** The presence of unsaturated hydrocarbon fatty acids and sterols prevents packing and enhances membrane fluidity, favoring the open state and functionality of aquaporins.

There is a general understanding that membrane AQP proteins diffuse laterally, associated with several layers of lipids, with the positions of the lipids in the first solvation shell being modulated also by irregularities in the protein interface. Therefore, the physical properties and chemical composition of the membrane will influence the functionality of the AQPs and could regulate their open/closed state (**Figure 2**). Also, it is becoming increasingly clear that AQP/AQP and AQP/protein interactions represent an important coordination system involved in cellular functions and need to be elucidated.

Another aspect to consider is the mechanical or therapeutic/food uses of vesicles rich in AQPs. Filters with a tremendous potential use in engineering, for water filtration, are being developed. The therapeutic/food aspect has been initiated and will be a very exciting line of plant AQPs exploitation in the future (Martínez-Ballesta et al., 2016). Furthermore, one of the challenges for the next 10 years is to use widely the expected increase in computational resources, due to the ongoing development of both hard- and software, to be able to implement the anticipated possibility of simulating both millisecond events, with all atom models, and truly macroscopic time scales, for simplified models. With the increasing number of particles that can be simulated, we expect studies on membranes to become gradually more dominant. However, the progress that is being made in the investigation of lipid/peptide force fields and protein/protein interactions is still a prerequisite for this.

## Author Contributions

MM-B her contribution was reviewed the aspect of interactions of aquaporins with aquaporins in the membranes. MC her contribution was reviewed the aspects of interactions of aquaporins with lipids in the membranes. The completed manuscript was corrected as a whole.

## Conflict of Interest Statement

The authors declare that the research was conducted in the absence of any commercial or financial relationships that could be construed as a potential conflict of interest.
